# Classification in Early Fire Detection Using Multi-Sensor Nodes—A Transfer Learning Approach

**DOI:** 10.3390/s24051428

**Published:** 2024-02-22

**Authors:** Pascal Vorwerk, Jörg Kelleter, Steffen Müller, Ulrich Krause

**Affiliations:** 1 Faculty of Process- and Systems Engineering, Institute of Apparatus and Environmental Technology, Otto von Guericke University of Magdeburg, Universitätsplatz 2, 39106 Magdeburg, Germany; ulrich.krause@ovgu.de; 2 GTE Industrieelektronik GmbH, Helmholtzstr. 21, 38-40, 41747 Viersen, Germany; joerg.kelleter@gte.de (J.K.); steffen.mueller@gte.de (S.M.)

**Keywords:** multi-sensor nodes, early fire detection, gas sensors, transfer learning, electronic nose, feature fusion, linear discriminant analysis (LDA), classification

## Abstract

Effective early fire detection is crucial for preventing damage to people and buildings, especially in fire-prone historic structures. However, due to the infrequent occurrence of fire events throughout a building’s lifespan, real-world data for training models are often sparse. In this study, we applied feature representation transfer and instance transfer in the context of early fire detection using multi-sensor nodes. The goal was to investigate whether training data from a small-scale setup (source domain) can be used to identify various incipient fire scenarios in their early stages within a full-scale test room (target domain). In a first step, we employed Linear Discriminant Analysis (LDA) to create a new feature space solely based on the source domain data and predicted four different fire types (smoldering wood, smoldering cotton, smoldering cable and candle fire) in the target domain with a classification rate up to 69% and a Cohen’s Kappa of 0.58. Notably, lower classification performance was observed for sensor node positions close to the wall in the full-scale test room. In a second experiment, we applied the TrAdaBoost algorithm as a common instance transfer technique to adapt the model to the target domain, assuming that sparse information from the target domain is available. Boosting the data from 1% to 30% was utilized for individual sensor node positions in the target domain to adapt the model to the target domain. We found that additional boosting improved the classification performance (average classification rate of 73% and an average Cohen’s Kappa of 0.63). However, it was noted that excessively boosting the data could lead to overfitting to a specific sensor node position in the target domain, resulting in a reduction in the overall classification performance.

## 1. Introduction

The advantages of multi-sensor approaches to early fire detection over traditional smoke detectors have been extensively discussed in the previous literature [1,2,3]. The main advantages are improved coverage of the detection area [4], a shorter detection time [5,6,7], more accurate detection (improved sensitivity to real fires) [8,9,10,11] and a reduction in the false alarm rate [12,13].

In addition to the temporal and robustness aspects of early fire detection, the ability to differentiate between different types of fire scenarios can provide additional information to laypersons or first responders during alarms [14]. This can support effective identification and intervention, especially in the early stages of ongoing incipient fires where combustion products are barely visible [15].

Previous research has demonstrated the effectiveness of employing multi-sensor approaches to distinguish various fire materials based on their unique “odor prints” [16,17,18]. However, these studies faced limitations in their training and validation datasets. Some were confined to a single room setting [19], while others were constrained to a binary output (fire/no fire) when utilizing data from different environments [20,21].

Generally, fire events are infrequent occurrences throughout a building’s lifespan. The scarcity of real event data poses challenges and necessitates reliance on data obtained from experimental setups or simulations [22]. However, conducting such (large-scale) experiments is expensive, and the availability of large-scale test rooms is very limited [21]. Given these constrains, there is an urgent need to investigate the effective transfer of data from small-scale laboratory setups to real room applications.

In this work, we address the research question (RQ) of whether multi-sensor data generated in a small-scale laboratory setup can be used to identify various incipient fire scenarios in a large-scale room setup.

To our knowledge, existing transfer learning methodologies have not been employed in the field of early fire detection using multi-sensor nodes. Furthermore, it remains uncertain whether, in general, the differentiation of various incipient fire scenarios during their initial stages is achievable based on multi-sensor data.

In this study, we employed two primary methodologies from the transfer learning research domain. We leveraged both feature representation transfer and instance transfer in order to identify different incipient fire scenarios in a real EN54 standard test room, relying solely on training data generated in a small-scale laboratory setup. Subsequently, we assessed the classifier’s performance at various sensor node positions within a large-scale test room.

The novelty of this work lies in its approach to distinguish between various incipient fire scenarios in their initial phases using solely training data from a small-scale setup. Prior research has typically been confined to a single experimental setup for both model construction and testing, or it has been restricted to binary model prediction (fire/no fire), simplifying the classification problem and incurring high data generation costs. This study addresses two primary limitations in the existing literature. Firstly, we present a comprehensive workflow for cost-effective data acquisition and model development in the field of early fire detection employing multi-sensor nodes. Secondly, we apply this workflow to a multi-classification problem, for which we differentiate between four distinct fire scenarios in their earliest stages. Previous work has predominantly focused on simpler binary classification problems and more advanced fire scenarios where detection is generally more straightforward. The proposed approach provides valuable additional information about the nature of an ongoing incipient fire event, enabling first responders or firefighters to make more informed decisions, such as formulating intervention recommendations or enhancing situational awareness.

## 2. Related Work

Prior research has explored various methodologies for fire detection and identification using multi-sensor data.

Solórzano et al. [21] achieved a classification rate of approximately 68% using training and test data from normative test fires conducted in a standard EN-54 test room. The authors stated that the classification rate could be increased to 96% by incorporating additional training and test data from laboratory experiments. In their recent publication [20], Solórzano et al. corroborated these findings, reporting a classification rate ranging from 52% to 70% (or 88% with additional training and test data generated in a small-scale setup).

However, in both studies, the model output was confined to a binary prediction (fire/no fire), leading to a significantly simpler classification problem compared to our study. Additionally, the test data consistently encompassed data from the same room environment that had already been utilized for training the model.

Other studies, as summarized in [3], were also primarily constrained to a binary decision problem (fire/no fire) and/or confined to a single experimental environment.

Milke et al. [23] defined hard rules utilizing a sensor array comprising temperature, light obscuration, CO_2_, MOX and O_2_ sensors in order to distinguish between “flaming fire”, “smoldering fire” and “nuisance”. The authors attained a classification rate of 90% and could enhance the classification rate up to 97% by employing a three-layer neural network as the model instead of hard rules. However, the training and test data were derived from experiments conducted in the same test room.

Ni et al. [24] constructed a classification model to categorize various wire insulation materials (PVC, Teflon, Kapton and silicone rubber) based on the volatiles released during electrical overload. The authors employed dimension reduction (PCA) and a K-NN classifier as the classification model and achieved a classification rate of up to 82% for four different classes. However, the training and test data were derived from the same experimental setup using the leave-one-out method.

Experiments in prior studies primarily utilized standard test fires, resulting in considerably higher emissions and, consequently, clearer sensor signals. In contrast, our study encompasses the initial phases of ongoing incipient fires within the experimental setup. Moreover, previous studies often focused on binary or ternary classification problems, with Ni et al. [24] being a notable exception. Another limitation in previous research is the generation of training and test data within the same experimental environment, which poses a constraint for real-world applications. The novelty of our work lies in utilizing data from two distinct experimental environments.

### 2.1. Early Fire Indicators

Previous studies have employed various combinations of multi-sensor measurements for early fire detection. Solórzano et al. [20] utilized hydrogen (H_2_), methane (CH_4_), nitrogen oxides (NO_*x*_) and volatile organic compounds (VOCs) in a multi-sensor array. The authors emphasized the significance of CO and VOCs as early fire indicators due to their substantial emissions during incipient fire scenarios such as smoldering fires. Nazir et al. [25] corroborated these findings by including air temperature, humidity, CO_2_ and ammonia (NH_3_) in their study.

Krüger et al. [26] and Hayashi et al. [27] identified substantial releases of H_2_ during the smoldering process of various polymeric materials commonly present in households such as wood, PUR foam and PE. The authors concluded that H_2_ can serve as an early fire indicator that precedes the substantial emissions of CO and smoke.

Gutmacher et al. [28] corroborated these findings, emphasizing that CO and H_2_ are the most crucial gases for detecting the early stages of smoldering fires.

In our previous study [29], we validated these observations. We examined particulate matter (PM), VOCs, CO, CO_2_, H_2_, ultraviolet radiation (UV), air temperature and humidity as early fire indicators during different incipient fires conducted in a standard EN 54 test room. By varying the distance between the sensor node and the fire source, we identified five significant early fire indicators: H_2_, CO, PM0.5 (PM < 0.5 μm), PM1.0 (0.5 μm < PM < 1.0 μm) and VOC.

### 2.2. Transfer Learning

Weiss et al. [30] emphasized the challenges in obtaining training and test data from the same domain for real-world machine learning applications, particularly in cases where data collection is impractical due to high costs or difficulty. This challenge is particularly relevant in the context of (early) fire detection using multi-sensor nodes, where generating data in real room setups is prohibitively expensive and the availability of fire test rooms is extremely limited. The authors emphasize the importance of employing less expensive training data from a different domain for model building. This concept is known as transfer learning.

Zhuang et al. [31] defined transfer learning as the enhancement of a target learner using knowledge from a “[…] different but related” [31] source domain. The primary objective is to decrease reliance on (expensive) data from the target domain.

According to Kim et al. [32], transfer learning aims to learn a target predictive function $f_{T}\left(\cdot \right)$ from pairs $\left\{x_{i},y_{i}\right\}$ generated in a source domain $D_{S}$, where $x_{i}\in X$ and $y_{i}\in Y$. In the subsequent work, the notation provided by Kim et al. [32] given in Table 1 is adopted, with the index *S* representing the source domain $D_{S}$ and the index *T* representing the target domain $D_{T}$.

According to Cook et al. [33], a certain relationship must exist between $D_{S}$ and $D_{T}$ in order to be able to transfer knowledge from $D_{S}$ to $D_{T}$. In our case, the feature space in both $D_{S}$ and $D_{T}$ is essentially the same (sensors, and selected sensor measurements are identical), thus satisfying Equation (1).

(1) $$X_{S}=X_{T}$$

However, the scaling and rotation of the feature spaces $X_{S}$ and $X_{T}$ differs slightly due to the distinct room settings.

In these feature spaces $X_{S}$ and $X_{T}$, the marginal probability distribution P(X) is not equal because the “activity” in $D_{S}$ and $D_{T}$, respectively, is not exactly the same (the experiments in $D_{S}$ are downscaled; see Section 3.2). This assumption is given in the following Equation (2).

(2) $$P\left(X_{S}\right)\neq P\left(X_{T}\right)$$

In this work, the label space Y in $D_{S}$ and $D_{T}$ is identical, as we conducted the same types of fire experiments in both domains (see Section 3.2), as given in Equation (3).

(3) $$Y_{S}=Y_{T}$$

As the objective prediction function f(·) is defined as f(·)=P(y|x) and P(X) varies between $D_{S}$ with respect to $D_{T}$ (see Equation (2)), f(·) differs for $D_{S}$ and $D_{T}$, as shown in Equation (4).

(4) $$f_{S}\left(\cdot \right)\neq f_{T}\left(\cdot \right)$$

This finally results in a different task T to learn, so that

(5) $$T_{S}\neq T_{T}$$

Cook et al. [33] defined two primary types of transfer learning approaches to address disparities between $D_{S}$ and $D_{T}$.

The first approach is feature representation transfer, which aims to mitigate the differences between the feature spaces $X_{S}$ and $X_{T}$. According to Cook et al. [33], feature representation transfer is typically achieved by mapping both $X_{S}$ and $X_{T}$ to a new feature space X through functions $g:X_{S}\to X$ and $f:X_{T}\to X$. Dimension reduction is a commonly employed technique in this context [33].

The second transfer learning approach is instance transfer, where a small amount of data from the target domain is utilized to weight instances from the source domain. Since this approach works particularly well under the condition of equivalent feature spaces $X_{S}$ and $X_{T}$, instance transfer is typically applied after feature representation transfer [33]. A common method for instance transfer is the TrAdaBoost algorithm proposed by Dai [34], which has already been employed in combination with an SVM classifier to categorize atmospheric dust aerosol particles in a transfer learning application [30].

## 3. Materials and Methods

### 3.1. Sensor Nodes

**Figure 1:**
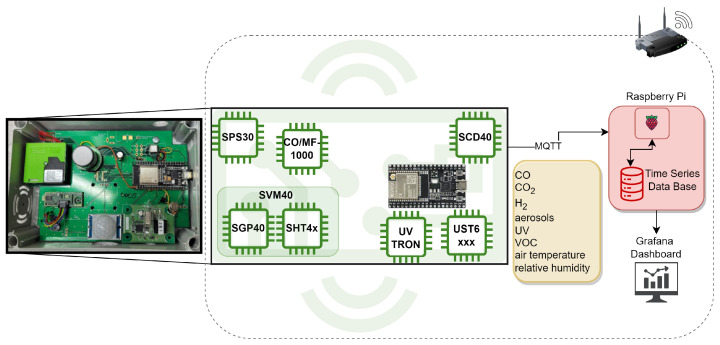
Sensor node with multiple sensors and data transfer via MQTT to Raspberry Pi.

We employed muti-sensor nodes for data collection, as illustrated in Figure 1. Each sensor node was equipped with sensors, including an SPS30, SGP40, SHT4x, CO/MF-1000, UST6xxx and SCD40, that measured parameters such as PM, VOC, relative air temperature, air humidity, CO, H_2_ and CO_2_.

The sensors on each sensor node were controlled by a microcontroller (ESP32). Communication between the microcontroller and the broker/server (Raspberry Pi) was via WiFi using the MQTT protocol. The microcontroller sent sensor data in JSON format to the Raspberry Pi, where a Python script decoded the information and recorded it in an Influx time series database. The database automatically assigned an unique UTC timestamp to each measurement vector.

For real-time monitoring during the experiments, a Grafana dashboard was utilized. Data were exported from the Influx time series database as a CSV file using a Python script. Each sensor node in the network was equipped with the sensors listed in Table 2.

A consistent sampling rate of one sample per 10 s was maintained throughout all experiments. This decision was influenced by the characteristics of the sensors in use. Specifically, the CO/MF-1000 sensor had a T90 response time of approximately 25 s: capturing 90% of the gas concentration within this time frame [35]. Likewise, the UST6xxx sensor relied on internal temperature cycles with a 10 s interval for H_2_ detection [36]. Hence, opting for a sampling rate exceeding one sample per 10 s would not yield any additional information.

To minimize cross-sensitivity between CH_4_, CO and alcohol, we selected the UST6xxx sensor containing the GGS 6530 T gas sensing element. The UST6xxx exhibits nearly no response to CH_4_ exposure up to 1000 vppm, and it sustains this characteristic at a heating temperature of 475 °C [36].

### 3.2. Experiments and Datasets

**Figure 2:**
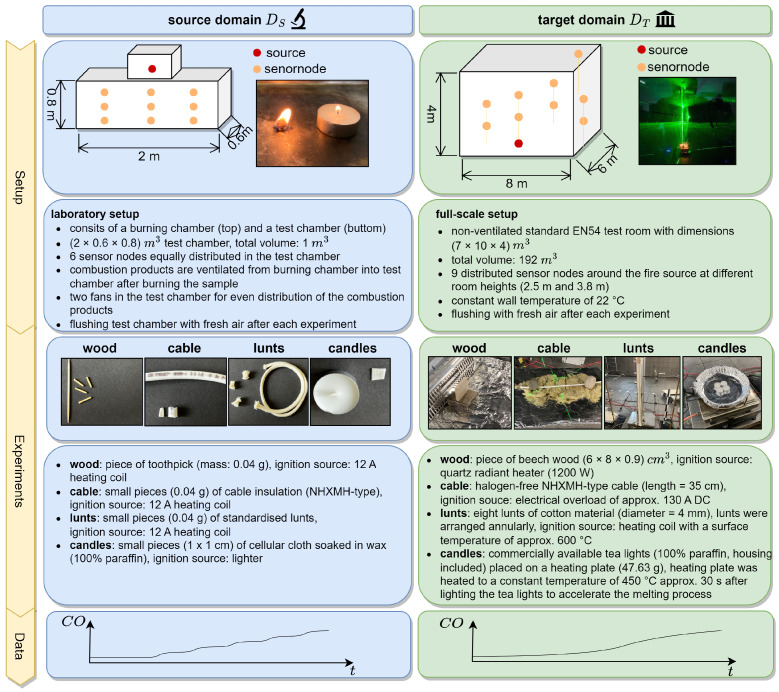
Experimental setup in $D_{S}$ (**left**) and $D_{T}$ (**right**); 4 different incipient fire experiments: smoldering wood, smoldering cable, glowing lunts and candle fire.

Following the idea of transfer learning discussed in Section 2.2, we used two experimental setups in order to represent the source domain $D_{S}$ and the target domain $D_{T}$. The two setups are exemplarily shown in Figure 2.

A (2 × 0.6 × 0.8) m^3^ test chamber served as the small-scale setup (source domain $D_{S}$), and we exposed six sensor nodes to various fire loads using cotton, cable insulation, candle wax and wood (see Figure 2, left). This experimental setup was used to generate the source domain dataset (ds_dataset).

**Figure 3:**
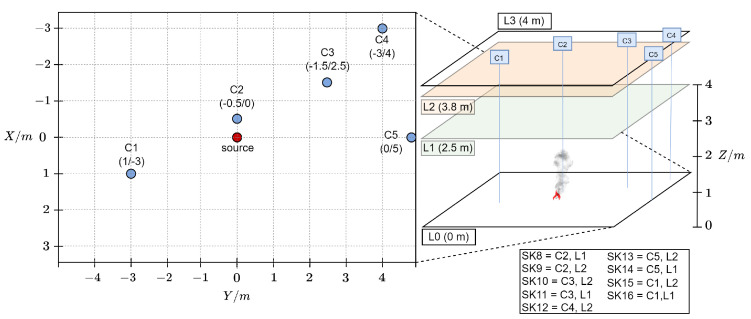
Sensor node positions in $D_{T}$; L0 = ground layer, L1 = height at 2.5 m, L2 = height at 3.8 m, C1–C5 = chains containing two sensor nodes (one at L1 and one at L2), and SK8–SK16: unique sensor node IDs.

An unventilated standard EN54 test room with dimensions (7 × 10 × 4) m^3^ was used as the large-scale setup (target domain $D_{T}$) to generate the target domain dataset (dt_dataset). The fire source was positioned in the center of the room. Nine distributed sensor nodes were placed around the source as shown in Figure 3.

In both domains, four distinct fire types—wood, cable, lunt and candle fires—were executed. Table 3 provides a summary of the burning material mass, repetitions, stages and ignition source type. A more comprehensive description of the experiments conducted in the target domain $D_{T}$ is given in [29].

The experiments conducted in $D_{S}$ represent scaled-down setups of the experiments performed in $D_{T}$. For equivalence, we employed identical materials in both domains but adjusted the mass of the burning material and the combustion process as follows.

To represent the smoldering wood fire, we used small pieces of toothpick. The toothpicks were standardized, and the mass of one piece of toothpick was 0.04 g. A DC heating coil (12 A) was used as the ignition source in order to ensure non-flaming combustion. The heating coil was a 1 mm-thick constantan wire twisted into a spiral consisting of 15 windings and an inner diameter of 100 mm.

The cable fire was simulated using small pieces (0.04 g) of the same cable insulation material used in $D_{T}$. As with the wood scenario, the 12 A DC heating coil was used as the ignition source.

The lunt fire was scaled down equivalently by using small pieces (0.04 g) of the lunts used in $D_{T}$. The ignition source was again the 12 A DC heating coil.

Downscaling of the candle fire was not trivial, as the wax fire produces high flames even with smaller amounts of wax material. To control the size of the flame, we used small pieces of cotton that were soaked in wax. The cotton acted as a wick. Its surface size served as the controlling parameter for the size of the flame.

As depicted in Figure 2, variations were observed in the temporal increase of sensor measurements in $D_{S}$ and $D_{T}$. This aligns with the findings reported by Solórzano et al. [21].

This contrast can be attributed to two primary factors. Firstly, there is a significant difference in the propagation behavior in $D_{T}$ with respect to $D_{S}$ due to the size of the room and the ventilation conditions. In $D_{S}$, the combustion products exhibit nearly uniform distribution due to static ventilation and the small room size. In contrast, the propagation behavior in the non-ventilated $D_{T}$ is predominantly influenced by agglomeration and gravitational settling [29].

Secondly, the combustion undergoes variations over time as a consequence of the downscaling of the sample size in $D_{S}$. The sub-processes of the combustion process, including heating, release of pyrolysis gases, smoldering and glowing, take place at considerably shorter time intervals in $D_{S}$ due to the small sample sizes.

We simulated various intensity levels that may occur in $D_{T}$ by accumulating the combustion products from multiple experimental stages in the test chamber in $D_{S}$. Consequently, we excluded the temporal component from our data and focused on the absolute values of the sensor measurements in the transfer learning approach, as described in more detail in Section 3.3 following.

The resulting datasets, ds_dataset and dt_dataset, underwent a data pre-processing step to achieve balance by randomly down-sampling to the minority class in order to avoid implicit class weights. After data balancing, the ds_dataset (training dataset) contained 770 datapoints per class and the dt_dataset (validation and boost dataset) contained 432 datapoints per class and sensor node position.

### 3.3. Methodology

**Figure 4:**
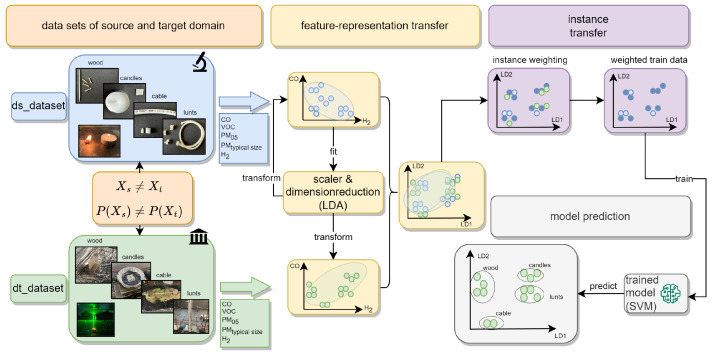
Methodology workflow; (1) data generation, (2) feature representation transfer using LDA, (3) instance transfer (weighting of training data) and (4) model building.

As proposed by Cook et al. [33], we applied both feature representation transfer and instance transfer in our study. The aim was to investigate the suitability of these two methods for classification in early fire detection considering the challenge of limited or no access to extensive data from large-scale experiments during model development. The overall workflow of data generation and processing is illustrated in Figure 4.

#### 3.3.1. Feature Representation Transfer

Linear Discriminant Analysis (LDA) was employed as a supervised dimension reduction method in the feature representation transfer step. LDA aimed to extract crucial information (reduced features) that are most relevant for distinguishing between fire scenarios based on data from $D_{S}$. As outlined in Section 2.1, the original input features for the LDA comprised CO, H_2_, VOC and PM (PM0.5 and PM1.0).

Both LDA and the scaler (min–max scaler with bounds [0, 1]) were applied to the data from $D_{S}$. The resulting transformation parameters were then utilized to transform the data in both $D_{S}$ and $D_{T}$ into the new feature space. Subsequently, the transformed data were employed to train a support vector machine (SVM) classifier using the transformed data from $D_{S}$, and its performance was validated at various sensor node positions in $D_{T}$.

#### 3.3.2. Instance Transfer

In addition to feature representation transfer, we implemented instance transfer using the TrAdaBoost algorithm presented in [34]. TrAdaBoost is a supervised domain adaptation method that utilizes limited data from $D_{T}$ to adjust a pre-trained model to new data: specifically, the target domain $D_{T}$ in our case [34]. The fundamental concept of TrAdaBoost is to adapt the knowledge learned from $D_{S}$ and apply it to a slightly different $D_{T}$, assuming that labeled data from $D_{T}$ are generally rare.

By definition, this approach requires the availability of limited instances from the target domain, which are employed to re-weight the training instances from $D_{S}$.

In practical terms, the target domain data for TrAdaBoost could be sourced from an actual fire event occurring in $D_{T}$ during the operation of the fire detection system or from a small number of large-scale experiments. Consequently, this method serves as a means to adapt the fundamental model trained on laboratory data to real-world application environments.

The objective of this study was to investigate how the performance of a classifier trained solely on laboratory data (ds_dataset) can be enhanced by incorporating small amounts of available data from $D_{T}$. To achieve this, we utilized from 1% up to 30% of the dt_dataset to re-weight the source domain instances using TrAdaBoost. Higher proportions of $D_{T}$ instances were employed to identify overfitting boundaries during the instance transfer step.

## 4. Results

This section is structured as follows. First, Section 4.1 presents the performance of the boosted model independent of the sensor node position in $D_{T}$. This means that instance transfer (boosting) was executed using data from the same sensor node position in $D_{T}$ as utilized for validation.

In Section 4.2, the boosting data were selected from a fixed sensor node position, and the model’s performance was subsequently validated across all sensor node positions in $D_{T}$ to identify potential overfitting effects based on the amount of boosting data taken from a specific sensor node position.

Beyond the performance assessments using various boosting strategies, the model was validated without any boosting; this served as the baseline for performance. This implies that only the feature representation transfer described in Section 3.3.1 was performed before applying the model to the $D_{T}$ data. This baseline performance facilitates the assessment of performance improvement when employing additional boosting strategies.

The Manhattan distance between the sensor node and the fire source in $D_{T}$ was employed to arrange different sensor node positions along the x-axis.

To enable performance comparisons across different models, the classification rate (average accuracy) was used as our primary performance metric. According to [37], the average accuracy for a multi-class classification problem is defined as shown in Equation (6).

(6) $$\frac{\sum_{i=1}^{l}\frac{tp_{i}+tn_{i}}{tp_{i}+fn_{i}+fp_{i}+tn_{i}}}{l}$$

Since we considered a balanced dataset for model validation, the classification rate is a suitable performance measure [37].

To compare the performance of the baseline model (non-boosted, only trained on ds_dataset) with a model that randomly assigns labels based on the given class distribution, we utilized Cohen’s κ as an additional performance metric, as suggested by Artstein et al. [38]. Cohen’s κ is a scaled value in the range of [−1, 1] that evaluates the model’s classification accuracy against the accuracy achieved by random label assignment according to a specified class distribution [38].

### 4.1. Classification Performance Independent of the Node Position

Table 4 shows the results of the baseline model only trained on the ds_dataset in terms of precision, recall, F1 score, classification rate and Cohen’s κ.

The baseline model exhibits its lowest performance at sensor node positions close to the wall—specifically, at sensor node 13 (global minimum, classification rate of 53%) and sensor node 14 (local minimum, classification rate of 58%)—in the dt_dataset, as shown in Table 4. This implies that the most significant difference between our laboratory setup ($D_{S}$) and $D_{T}$ occurs at positions close to the wall in $D_{T}$.

The Cohen’s κ ranges from 0.36 (minimum at test sensor node 13) up to 0.58 (maximum at test sensor node position 08). According to Landis et al. [39], this can be categorized as “fair” (0.2 ≤ κ ≤ 0.4) to “moderate” (0.4 ≤ κ ≤ 0.6) model performance.

To adapt the model derived from $D_{S}$, additional model boosting was performed. Initially, boosting was performed assuming knowledge about the distance between the sensor node and the fire source in $D_{T}$.

**Figure 5:**
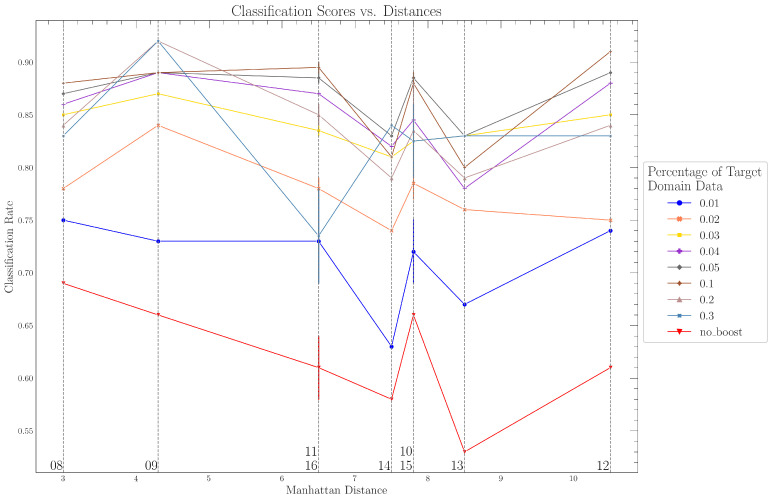
Classification rate using instance weighting (boosting) based on test position.

Figure 5 shows the classification rate as a function of the sensor node position used for boosting and testing. The different lines represent the amount of data used for boosting (1% to 30%) from the test position in $D_{T}$. The “no_boost” line represents the classification performance of the baseline model.

It can be seen from Figure 5 that the classification rate of the non-boosted baseline model ranges from a global minimum (classification rate of 53% at sensor node position 13) up to a global maximum at sensor node position 08 (classification rate of 69%; see also Table 4). There is a continuous decrease in the classification rate from the lowest Manhattan distance of 3.0 m (sensor node position 08) to a Manhattan distance of 7.5 m (sensor node position 14). The classification rate then reaches a local minimum of 58% at sensor node position 14. A local maximum with a 66% classification rate can be observed at sensor node positions 10 and 15. Moving to the next-higher Manhattan distance (sensor node position 13), the classification rate reaches a global minimum of 53%. Sensor node position 12 (highest Manhattan distance to the source) shows a classification rate of 61%, which is 3% more than the local minimum at sensor node position 14.

Looking at the different boosting curves (0.01 to 0.3) in Figure 5, it is evident that additional information from $D_{T}$ used for model boosting cannot completely offset the local minima in the classification rate at sensor node positions 13 and 14 close to the wall. The previously described trend in the classification rate remains essentially the same. However, differences in the classification rate between different sensor node positions (except for sensor nodes 13 and 14 close to the wall) can be mitigated by using additional boosting, particularly for boost amounts up to 5%. Although higher boost amounts (from 10% to 30%) lead to a global maximum of the classification rate (sensor node position 09, 20% boosting data), the differences in the classification rates between different sensor node positions increase compared to boost amounts of around 5%.

Nevertheless, the differences between the sensor node positions (except for the positions close to the wall) are increasingly compensated for by additional boosting. This implies that the model trained only based on $D_{S}$ can be adapted to a new environment with small amounts of available data from $D_{T}$.

**Figure 6:**
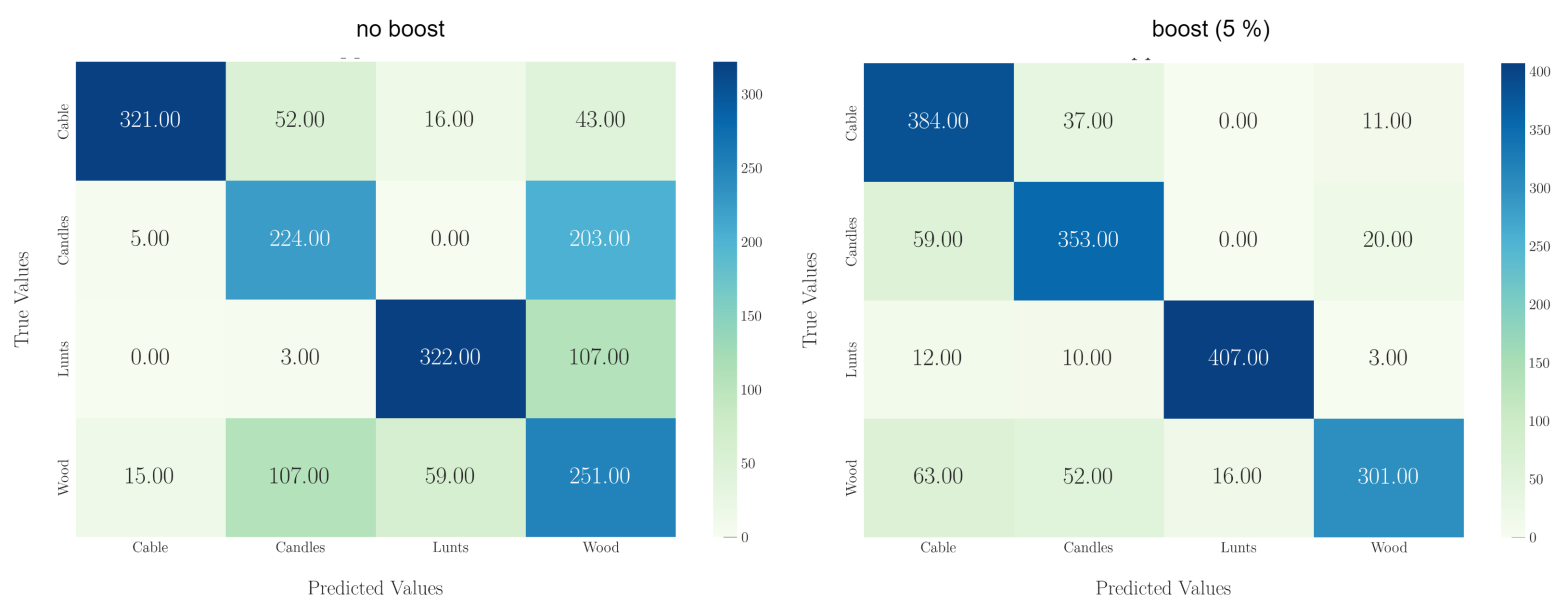
Comparison of confusion matrices for non-boosted case (**left**) and boosted case (**right**) for sensor node position 8 in $D_{T}$.

Figure 6 following illustrates the model’s performance for sensor node position 8 in $D_{T}$ for the non-boosted model (left) and the boosted model (boosted with 5% of the data from sensor node 8).

It can be seen from Figure 6 that the baseline model only trained on data from $D_{S}$ primarily misclassifies between the candle scenario and the wood scenario. This misclassification can be attributed to the experimental procedure. In $D_{S}$, we employed small pieces of cellular cloth soaked with candle wax to represent the candle wax fire in a small-scale test. However, when the wax was fully burned, the cellular cloth (wick) started to glow and smolder at the end of each experiment. Since this combustion process closely resembles the glowing process of wood, it likely led lead to misclassification between the wood and candle fires.

Comparing the wick size to the mass of wax in $D_{S}$ and $D_{T}$, the ratio is considerably higher in $D_{S}$ than in $D_{T}$. As discussed in Section 3.2, scaling down a wax fire is challenging. To regulate the flame size of the wax fire, we had to use a much higher ratio of wick volume to wax volume. The volume of the wick compared to the volume of the burning wax was negligible in $D_{T}$. Consequently, fewer smoldering or glowing artifacts were observed in the dt_dataset than in the ds_dataset, resulting in the aforementioned misclassification.

This misclassification was evident at other test sensor node positions in $D_{T}$. Figure 6 (right) illustrates that the misclassification can be minimized by employing additional boosting.

Table 5 provides an overview of the average model performance across all sensor node positions in $D_{T}$, represented by the mean classification rate and the mean Cohen’s κ for different boosting scenarios (ranging from no boosting to 30% boosting data).

It can be seen from Table 5 that the mean model performance (mean classification rate and mean Cohen’s κ) generally improves with model boosting. The model performance increases with an increasing amount of boosting data and reaches its maximum (87% mean classification rate and a mean Cohen’s κ of 0.83) at 5% (up to 10%) of boosting data. The Cohen’s κ ranges from 0.49 (“moderate”) up to 0.83 (“perfect”) according to Landis et al. [39].

As the amount of boosting data increases, the average model performance decreases, although it remains higher than the model performance without boosting. This phenomenon has already been discussed based on Figure 5. Even though higher amounts of boosting data lead to global maxima for the classification rate, the difference in the classification rate increases, causing the mean classification rate to decrease.

In summary, we found higher classification rates with boosting compared to the no-boost baseline model. The sensor node positions close to the wall show a local minimum of the classification rate regardless of the model used (no boost vs. different amounts of boosting data).

### 4.2. Classification Performance Dependent of the Sensor Node Position

**Figure 7:**
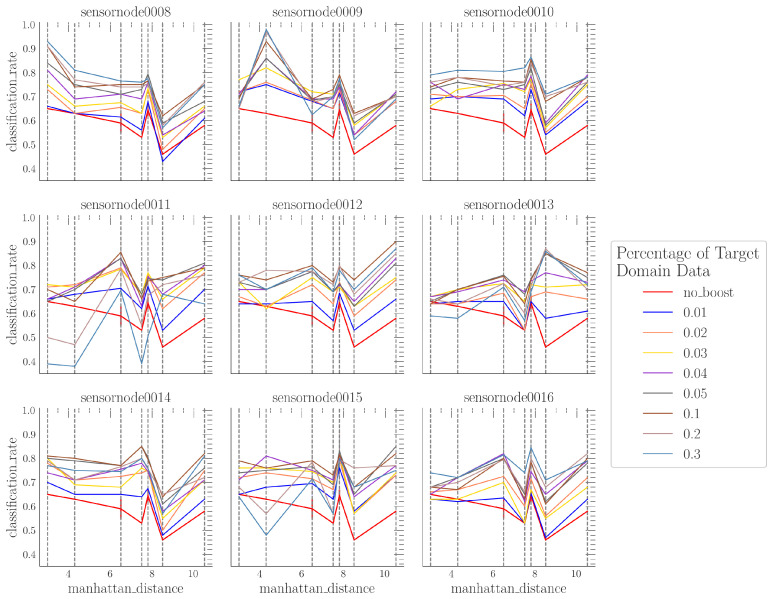
Classification rate using instance weighting (boosting) and random sensor node positions.

The results presented in Section 4.1 represent an optimal boosting scenario with respect to the distance between the sensor node and the fire source. The data used for boosting were derived from the same sensor node position used for testing without utilizing the validation data already employed for boosting from the dt_dataset. However, in a real-world application, the distance between the sensor node and the fire source will be unknown. To investigate this scenario, we utilized boosting data from one fixed sensor node position and evaluated the model performance across all sensor node positions. The results are shown in Figure 7.

The red line in Figure 7 represents the baseline model performance without model boosting. The sub-figures are labeled based on the sensor node position used for boosting. We utilized the same amount of boosting data as in Section 4.1 (1% to 30%).

We observed that the global maximum of the classification rate was reached when the test sensor node position and the sensor node position used for boosting were the same (e.g., see sub-figure “sensornode0009” at the Manhattan distance of sensor node position 09 in Figure 7). However, it can be seen from Figure 7 that the model performance at test sensor node positions different from the boosting sensor node achieves higher classification rates compared to the baseline model (no boosting). This holds true for boosting data amounts up to 5%, while higher amounts of boosting data from a particular sensor node position lead to overfitting to the boosting sensor node position. This effect is visible in Figure 7 when the classification rate of the boosted model falls below the baseline classification rate.

Another observation from Figure 7 is that there is still a local minimum in the classification rate at sensor node positions 13 and 14 (positions close to the wall). However the difference between $D_{S}$ and $D_{T}$ can be compensated for (see sub-figures “sensornode0013” and “sensornode0014” in Figure 7) if data from these sensor node positions are used for model boosting.

Table 6 shows the mean classification rates and the mean Cohen’s κ values over all sensor node positions used for testing and boosting as a function of the amount of boosting data (0–30%).

It is essential to emphasize that the mean performance measure represents the static boost scenario (boosting data were taken from only one sensor node position in $D_{T}$, and the model was then tested on all sensor node positions in $D_{T}$).

Table 6 indicates that the mean classification rate, as well as the mean Cohen’s κ, is significantly higher when using additional boosting compared to the cases without any boosting (no_boost). Furthermore, it can be observed that the performance increases with the amount of boosting data used, up to the maximum performance at 5%.

At higher amounts of boosting data, the average performance decreases again due to increased overfitting to individual sensor node positions. At 30% boosting, the average performance in terms of mean classification rate and mean Cohen’s κ is comparable to the average performance without boosting.

## 5. Discussion

As highlighted by Burgués et al. [40], a common challenge in machine-learning-based prediction lies in the limitations of examples available in the training data.

In this study, we considered four different incipient fire scenarios that have been identified as the main initial fire sources in historic and cultural buildings in Germany [41]. However, the model’s predictive accuracy may be compromised in the presence of different or additional burning materials (or superpositions of different materials) that have not been accounted for in this study.

Nevertheless, our research demonstrates the feasibility of classifying various incipient fire scenarios using multi-sensor training data from a small-scale setup. This opens up the possibility of generating cost-effective and extensive data for other burning materials that encompass different combustion conditions and/or superpositions with different nuisance scenarios (such as deodorant, dust, etc.).

Burgués et al. [40] also highlighted the model’s limitation to a specific range of tested (in their case, odor) concentrations. In our study, we focused on early phases of incipient fires, which are primarily characterized by the combustion process and the masses of burning material relative to the room volume. From our current results, we cannot extrapolate the model performance to more advanced stages of the conducted fire scenarios. Different combustion conditions result in the distinct release of combustion products over time. However, the experimental setup presented for $D_{S}$ enables the generation of data for these diverse combustion conditions, including those of more advanced courses of various fire scenarios.

Another consideration is that we did not include test positions where the sensor node is positioned very close to the fire source in $D_{T}$. This might lead to significantly different sensor signals due to sensor override. In such cases, deterioration in model performance would be expected.

We found that the baseline model trained only on $D_{S}$ data tends to misclassify between the candle and the wood fire scenarios. As discussed in Section 4.1, this misclassification can be attributed to the experimental setup used for the candle fire in $D_{S}$. In further experiments, it would be advisable to alter the wick material to a non-combustible substance to minimize glow and smolder effects. Alternatively, stopping the experiment before complete wax combustion could prevent glow and smolder artifacts in the data. In the small-scale setup ($D_{S}$), we used a fan to transport combustion products from the combustion chamber into the test chamber where the sensor nodes were located. The uncertainty regarding when combustion products from the smoldering wick entered the test chamber makes it challenging to remove these artifacts from the ds_dataset afterward.

When comparing the classification performance of our study with previous research, it is noteworthy that our non-boosted model already achieves comparable results (classification rate up to 69%) compared to studies such as Solórzano et al. [20] (52% to 70%). With additional boosting, the model performance can be further increased up to 87%: yielding results comparable to [20] with additional laboratory data (88%) or only slightly lower performance than in [23] (90%). It is essential to recognize that, unlike previous research, we addressed a four-class classification problem and employed two distinct experimental settings to generate the test and training data, thereby limiting direct comparisons.

In comparison with a similar classification problem ([24]), our boosted model achieves an average classification rate that surpasses Ni et al.’s [24] result (82% classification rate) by 5%. It is important to note that Ni et al. utilized a single experimental setup to generate the training and test data.

Another limitation to comparing our model’s performance with previous studies is our consideration of performance across various sensor node positions. It is evident that positions with lower classification rates will adversely affect the average model performance. The previous literature did not account for position-dependent performance measures, which hold great relevance in practical applications. Hence, it can be assumed that the performance comparison of our model with the previous work leans towards the conservative side.

Drawing from the outcomes presented in this study, we posit that the introduced approach, which combines transfer learning methods with multi-sensor data, is promising and highly relevant for the practical application of data-driven models relying on multi-sensor data. For instance, cost-effective generation of data for various fire materials or combinations can be accomplished on a small laboratory scale, including overlays with nuisance variables, to facilitate the early detection of fires in real room environments.

The demonstrated approach can be expanded to diverse application domains. For instance, investigating outdoor applications such as forest fire detection or air monitoring in industrial plants is a plausible direction for future investigations. However, outdoor environments exhibit distinct ventilation conditions, characterized by the formation of plumes, and a reduced tendency for the accumulation of combustion products. In scenarios like forest fire detection, combustion products tend to accumulate beneath the canopy or due to atmospheric inversion, leading to substantial influence of environmental conditions on the propagation behavior of combustion products.

The misclassification between candles and wood highlights that similar combustion processes lead to lower classification performance, particularly in the early detection phase. The classification rate of the non-boosted model (53% to 69%) indicates potential uncertainty in the classification, which should not be underestimated, especially during the initial stages of incipient fires. We presume that the classification rate might improve with more advanced fires that give clearer sensor signals. However, in an application scenario, an anomaly detector would be connected before the classifier to act as a trigger. One approach could involve using the time interval between the current classification and the triggering of the anomaly detector as a measure of the expected information quality of the classifier.

It is essential to acknowledge that, despite the approach presented in this work, the individual propagation behavior in the application room significantly influences the model’s performance. Notably, the model performance experienced a significant reduction at sensor node positions close to the wall in our study. A classification rate of 53% (sensor node position 13, non-boosted model) is relatively low even in a four-class classification problem and may result in misjudgment of the situation in a real application scenario. Since distance effects have not been considered in the previous literature when calculating performance measures, there is a pressing need for further research in this area. In general, a model can only recognize scenarios reliably if the sensor generates reliable input data. Future work should pay more attention to limitations associated with sensor positioning and incorporate these limitations into the evaluation process.

## 6. Conclusions and Outlook

This paper presents the results of employing two transfer learning methodologies—namely, feature representation transfer and instance transfer—within the context of early fire detection through multi-sensor nodes. The primary objective (RQ) of this study was to investigate whether multi-sensor data from a small-scale setup ($D_{S}$) can be used to classify various incipient fires in their early stages within an authentic room setting without the need to generate time- and cost-intensive data in large-scale setups.

In conclusion, we successfully generated multi-sensor data for four distinct types of incipient fires in a time- and cost-efficient manner within the small-scale experimental setup ($D_{S}$) outlined in this study. The $D_{S}$ data facilitated the extraction of crucial information to differentiate between various types of incipient fires. Based on this new feature space, a state-of-the-art classifier (SVM) was trained to classify unseen data from a large-scale setup.

We observed that the baseline model, trained exclusively on the $D_{S}$ data, consistently demonstrated the ability to classify four different incipient fire scenarios within $D_{T}$: achieving a classification rate of up to 69% and a Cohen’s κ of 0.58. However, the model’s performance is notably influenced by the distance between the sensor node and the fire source. In particular, we found that sensor node positions close to the wall exhibited lower classification performance (minimum classification rate of 53% and minimum Cohen’s κ of 0.36).

We identified that the decrease in performance primarily resulted from misclassification between the candle and wood scenarios. This misclassification was attributed to the experimental setup of the candle (wax) fire in $D_{S}$. In further investigations, we recommend optimizing the experimental setup to prevent the ds_dataset from acquiring glowing or smoldering artifacts. Based on our findings, we anticipate that such optimization will indeed enhance the performance of the baseline model.

Another finding of this study is that the model’s performance can be enhanced through additional model boosting (instance transfer), which is applicable when there is access to (small) amounts of real room data. However, it is crucial to keep the amount of boosting data low to avoid overfitting the model to a particular room situation or sensor node position. In our study, we determined the optimal amount of boosting data to be approximately 5% of the training instances in $D_{T}$.

In further research, we aim to extend the ds_dataset to include a broader range of combustible materials. Additionally, we plan to investigate superpositions of different combustible materials in $D_{S}$ and $D_{T}$. This is crucial to investigate, as real-world combustible objects often consist of mixtures of various materials. Another noteworthy aspect is the examination of superposition of nuisance scenarios with different fire scenarios, which will enhance the model’s robustness against side effects such as dust, humidity changes, etc.

To ensure a wider range of applications, future research should involve generating test data in diverse full-scale environments. This could encompass test rooms with varying geometries beyond the standard fire test room. Additionally, conducting full-scale outdoor tests would be valuable for extending the application of this concept to areas such as wildland fire detection or industrial facilities.

Another aspect to consider is that data processing, including the classification model presented in this study, is currently executed using the resources of the server (Raspberry Pi). In future research, we aim to explore the feasibility of conducting data processing directly on the ESP32. This would enhance the autonomy of the multi-sensor node, potentially reducing the notification time.

## Figures and Tables

**Table 1:** Transfer learning definitions and notations for source domain $D_{S}$ and target domain $D_{T}$ according to Kim et al. [32].

Notation Source Domain $D_{S}$	Notation Target Domain $D_{T}$	Description
$D_{S}=\left\{X_{S},P\left(X_{S}\right)\right\}$	$D_{T}=\left\{X_{T},P\left(X_{T}\right)\right\}$	domain
$X_{S}=x_{S1},\ldots ,x_{Sn}$	$X_{T}=x_{T1},\ldots ,x_{Tn}$	feature space
$P(X_{S})$	$P(X_{T})$	marginal probability distribution
$Y_{S}=y_{S1},\ldots ,y_{Sn}$	$Y_{T}=y_{T1},\ldots ,y_{Tn}$	label space
$f_{S}\left(\cdot \right)=P\left(y_{Si}|x_{Si}\right)$	$f_{T}\left(\cdot \right)=P\left(y_{Ti}|x_{Ti}\right)$	objective predictive function
$T_{S}=\left\{Y_{S},f_{S}\left(\cdot \right)\right\}$	$T_{T}=\left\{Y_{T},f_{T}\left(\cdot \right)\right\}$	task

**Table 2:** Overview of sensors in each sensor node.

Sensor	Manufacturer	Measurand	Unit
SPS30	Sensirion (Stäfa, Switzerland)	PM	cm^−3^
SGP40	Sensirion	VOC	A.U.
CO/MF-1000	MEMBRAPOR (Wallisellen, Switzerland)	CO	ppm
UST6xxx	UST (Sydney, Australia)	H_2_	ppm
SCD40	Sensirion	CO_2_	ppm
UVTRON	HAMAMATSU (Shizuoka, Japan)	UV photon	#
SHT4x	Sensirion	Temperature, Relative air humidity	°C, %

**Table 3:** Overview of the experiments carried out in $D_{S}$ and $D_{T}$.

Domain	Scenario	Mass	Stages	Repetitions	Material	Ignition Source
source	wood	0.04 g	5	4	beech wood	heating coil (12A)
	cable	0.04 g	5	6	cable isolation ^1^	heating coil (12A)
	lunts	0.04 g	6	4	cotton ^2^	heating coil (12A)
	candles	0.90 g	1	6	cellular cloth ^3^	lighter
target	wood	30.8 g	1	3	beech wood	quartz radiant heater ^4^
	cable	29.99 g	1	3	cable isolation ^1^	electrical overload ^7^
	lunts	100.20 g	1	3	cotton ^2^	heating coil ^5^
	candles	47.63 g	1	3	tea lights	heating plate ^6^

^1^ NHXMH-type; ^2^ manufacturer: “FehrErlen”; ^3^ soaked with candle wax (100% paraffin); ^4^ 1200 W, distance between heater and source = 5 cm; ^5^ surface temperature = 600 °C; ^6^ surface temperature = 450 °C; ^7^ 130 A DC.

**Table 4:** Precision, recall, F1 score, classification rate and Cohen’s κ for non-boosted model based on test sensor node position.

Test Sensor Node	Manhattan Distance [m]	Boost	Precision	Recall	F1 Score	Classific. Rate (Accuracy)	Cohen’s κ
08	3.0	no boost	0.74	0.68	0.69	0.69	0.58
09	4.3	no boost	0.69	0.64	0.65	0.66	0.53
10	7.8	no boost	0.69	0.64	0.65	0.66	0.54
11	6.5	no boost	0.67	0.56	0.58	0.58	0.43
12	10.5	no boost	0.68	0.60	0.62	0.61	0.47
13	8.5	no boost	0.60	0.51	0.52	0.53	0.36
14	7.5	no boost	0.66	0.56	0.58	0.58	0.42
15	7.8	no boost	0.71	0.64	0.66	0.66	0.53
16	6.5	no boost	0.69	0.63	0.64	0.64	0.51

**Table 5:** Mean classification rate and mean Cohen’s κ for different boosting strategies (dynamic boost).

Boost	Mean Classification Rate	Mean Cohen’s κ
no_boost	0.62	0.49
0.01	0.71	0.61
0.02	0.78	0.70
0.03	0.84	0.78
0.04	0.85	0.80
**0.05**	**0.87**	**0.83**
**0.10**	**0.87**	**0.83**
0.20	0.84	0.78
0.30	0.82	0.75

bold: maximum mean classification rate and Cohen’s κ for dynamic boosting.

**Table 6:** Mean classification rate and Cohen’s κ for different boosting strategies (static boost).

Boost	Classification Rate	Cohen’s κ
no_boost	0.62	0.49
0.01	0.67	0.56
0.02	0.69	0.58
0.03	0.71	0.61
0.04	0.72	0.62
**0.05**	**0.73**	**0.63**
0.10	0.71	0.61
0.20	0.67	0.56
0.30	0.64	0.51

bold: maximum mean classification rate and Cohen’s κ for static boosting.

## Data Availability

The used datasets can be provided on request.

## References

[1] McAvoy T.J., Milke J., Kunt T.A. (1996). Using multivariate statistical methods to detect fires. Fire Technol..

[2] Chen S.J., Hovde D.C., Peterson K.A., Marshall A.W. (2007). Fire detection using smoke and gas sensors. Fire Saf. J..

[3] Fonollosa J., Solórzano A., Marco S. (2018). Chemical Sensor Systems and Associated Algorithms for Fire Detection: A Review. Sensors.

[4] Rachman F.Z., Hendrantoro G., Wirawan A Fire Detection System Using Multi-Sensor Networks Based on Fuzzy Logic in Indoor Scenarios. Proceedings of the 2020 8th International Conference on Information and Communication Technology (ICoICT).

[5] Wu L., Chen L., Hao X. (2021). Multi-Sensor Data Fusion Algorithm for Indoor Fire Early Warning Based on BP Neural Network. Information.

[6] Liang Y.H., Tian W.M. Multi-sensor Fusion Approach for Fire Alarm Using BP Neural Network. Proceedings of the 2016 International Conference on Intelligent Networking and Collaborative Systems (INCoS).

[7] Nakıp M., Güzeliş C. Multi-Sensor Fire Detector based on Trend Predictive Neural Network. Proceedings of the 2019 11th International Conference on Electrical and Electronics Engineering (ELECO).

[8] Jana S., Shome S.K. (2023). Hybrid Ensemble Based Machine Learning for Smart Building Fire Detection Using Multi Modal Sensor Data. Fire Technol..

[9] Yu M., Yuan H., Li K., Wang J. (2023). Research on multi-detector real-time fire alarm technology based on signal similarity. Fire Saf. J..

[10] Gottuk D.T., Peatross M.J., Roby R.J., Beyler C.L. (2002). Advanced fire detection using multi-signature alarm algorithms. Fire Saf. J..

[11] Nakip M., Güzelíş C., Yildiz O. (2021). Recurrent Trend Predictive Neural Network for Multi-Sensor Fire Detection. IEEE Access.

[12] Milke J.A., Hulcher M.E., Worrell C.L., Gottuk D.T., Williams F.W. (2003). Investigation of Multi-Sensor Algorithms for Fire Detection. Fire Technol..

[13] Conrad T., Reimann P., Schutze A. A hierarchical strategy for under-ground early fire detection based on a T-cycled semiconductor gas sensor. Proceedings of the 2007 IEEE SENSORS.

[14] von der Linde M., Herbster C., Dobel C., Festag S., Thielsch M.T. (2023). Creating safe environments: Optimal acoustic alarming of laypeople in fire prevention. Ergonomics.

[15] Gutmacher D., Hoefer U., Wöllenstein J. (2012). Gas sensor technologies for fire detection. Sens. Actuators A Chem..

[16] Scorsone E., Pisanelli A.M., Persaud K.C. (2006). Development of an electronic nose for fire detection. Sens. Actuators B Chem..

[17] Fujinaka T., Yoshioka M., Omatu S., Kosaka T. Intelligent Electronic Nose Systems for Fire Detection Systems Based on Neural Networks. Proceedings of the 2008 The Second International Conference on Advanced Engineering Computing and Applications in Sciences.

[18] Joseph P., Bakirtzis D., Vieille A. (2015). An “electronic nose” as a potential device for fire detection of forest product fire loads in enclosures. Wood Mater. Sci. Eng..

[19] Andrew A.M., Shakaff A., Zakaria A., Gunasagaran R., Kanagaraj E., Saad S.M. Early Stage Fire Source Classification in Building using Artificial Intelligence. Proceedings of the 2018 IEEE Conference on Systems, Process and Control (ICSPC).

[20] Solórzano A., Eichmann J., Fernández L., Ziems B., Jiménez-Soto J.M., Marco S., Fonollosa J. (2022). Early fire detection based on gas sensor arrays: Multivariate calibration and validation. Sens. Actuators B Chem..

[21] Solórzano A., Fonollosa J., Marco S. (2017). Improving Calibration of Chemical Gas Sensors for Fire Detection Using Small Scale Setups. Proceedings.

[22] Kim Y.J., Kim H., Lee S., Kim W.T. (2021). Trustworthy Building Fire Detection Framework With Simulation-Based Learning. IEEE Access.

[23] Milke J.A. (1999). Monitoring Multiple Aspects of Fire Signatures for Discriminating Fire Detection. Fire Technol..

[24] Ni M., Stetter J.R., Buttner W.J. (2008). Orthogonal gas sensor arrays with intelligent algorithms for early warning of electrical fires. Sens. Actuators B Chem..

[25] Nazir A., Mosleh H., Takruri M., Jallad A.H., Alhebsi H. (2022). Early Fire Detection: A New Indoor Laboratory Dataset and Data Distribution Analysis. Fire.

[26] Krüger S., Despinasse M.C., Raspe T., Nörthemann K., Moritz W. (2017). Early fire detection: Are hydrogen sensors able to detect pyrolysis of house hold materials?. Fire Saf. J..

[27] Hayashi Y., Akimoto Y., Hiramatsu N., Masunishi K., Saito T., Yamazaki H., Nakamura N., Kojima A. Smoldering Fire Detection Using Low-Power Capacitive MEMS Hydrogen Sensor for Future Fire Alarm. Proceedings of the 2021 21st International Conference on Solid-State Sensors, Actuators and Microsystems (Transducers).

[28] Gutmacher D., Foelmli C., Vollenweider W., Hoefer U., Wöllenstein J. (2011). Comparison of gas sensor technologies for fire gas detection. Procedia Eng..

[29] Vorwerk P., Kelleter J., Müller S., Krause U. (2023). Distance-Based Analysis of Early Fire Indicators on a New Indoor Laboratory Dataset with Distributed Multi-Sensor Nodes. Fire.

[30] Weiss K., Khoshgoftaar T.M., Wang D. (2016). A survey of transfer learning. J. Big Data.

[31] Zhuang F., Qi Z., Duan K., Xi D., Zhu Y., Zhu H., Xiong H., He Q. (2021). A Comprehensive Survey on Transfer Learning. Proc. IEEE.

[32] Kim H.E., Cosa-Linan A., Santhanam N., Jannesari M., Maros M.E., Ganslandt T. (2022). Transfer learning for medical image classification: A literature review. BMC Med. Imaging.

[33] Cook D., Feuz K.D., Krishnan N.C. (2013). Transfer learning for activity recognition: A survey. Knowl. Inf. Syst..

[34] Dai W., Yang Q., Xue G.R., Yu Y. Boosting for transfer learning. Proceedings of the 24th International Conference on Machine Learning, ICML ’07.

[35] (2023). Carbon-Monoxide-Gas-Sensor_Datasheet. https://www.membrapor.ch/sheet/Carbon-Monoxide-Gas-Sensor-CO-MF-1000.pdf.

[36] (2023). DataSheet-GGS-6530-T_Rev2203. https://www.umweltsensortechnik.de/fileadmin/assets/downloads/gassensoren/single/DataSheet-GGS-6530-T_Rev2203.pdf.

[37] Sokolova M., Lapalme G. (2009). A systematic analysis of performance measures for classification tasks. Inf. Process. Manag..

[38] Artstein R., Poesio M. (2008). Inter-Coder Agreement for Computational Linguistics. Comput. Linguist..

[39] Landis J.R., Koch G.G. (1977). The Measurement of Observer Agreement for Categorical Data. Biometrics.

[40] Burgués J., Doñate S., Esclapez M.D., Saúco L., Marco S. (2022). Characterization of odour emissions in a wastewater treatment plant using a drone-based chemical sensor system. Sci. Total Environ..

[41] Kabat S. (2021). Brandschutz in Kirchen und Klöstern.

